# Nurses’ Knowledge and Attitudes Toward Pain Management at a Tertiary Hospital in Saudi Arabia: Impact of an Evidence-Based Instructional Program

**DOI:** 10.3390/healthcare14060729

**Published:** 2026-03-12

**Authors:** Mahmoud Abdel Hameed Shahin, Fatmah Alamoudi, Magda Yousif Ramadan, Adil Abdalla, Sarah Fahad Al Ojaimi, Nada Saleh Al Saadi, Anfal Shaheen Aleid, Hanan Alfahd

**Affiliations:** 1 Medical-Surgical and Critical Care Nursing, Nursing Department, Prince Sultan Military College of Health Sciences, Dhahran 34313, Saudi Arabia; mshahein@psmchs.edu.sa; 2 Medical Surgical Nursing, Nursing Department, Prince Sultan Military College of Health Sciences, Dhahran 34313, Saudi Arabia; aabdalla@psmchs.edu.sa; 3 Pediatric Nursing, Nursing Department, Prince Sultan Military College of Health Sciences, Dhahran 34313, Saudi Arabia; mramadaan@psmchs.edu.sa; 4 Nursing Department, Prince Sultan Military College of Health Sciences, Dhahran 34313, Saudi Arabia; sojaimi@psmchs.edu.sa; 5 Nursing Administration, King Fahad Military Medical Complex, Dhahran 34313, Saudi Arabia; hope_86@windowslive.com; 6 Nursing Education, King Fahad Military Medical Complex, Dhahran 34313, Saudi Arabia; a.aleid@modhs.med.sa (A.S.A.); hanan.fahd@kfmmc.med.sa (H.A.)

**Keywords:** pain management, nurses, knowledge, attitudes, educational intervention, Saudi Arabia

## Abstract

**Highlights:**

**What are the main findings?**

- Nurses demonstrated moderate baseline knowledge and generally positive attitudes toward pain management at a tertiary military hospital in Saudi Arabia.
- A 3 h evidence-based educational program produced statistically significant improvements in both knowledge and attitudes, with a moderate positive correlation between the two before and after the intervention.

**What are the implications of the main findings?**

- Short, targeted, evidence-based training can be used as a practical strategy to strengthen nurses’ pain management competence and reinforce patient-centered pain care in tertiary settings.
- Embedding ongoing pain education within hospital professional development may help sustain improvements and reduce persistent barriers (e.g., misconceptions around analgesics), supporting safer, more consistent pain assessment and management.

**Abstract:**

**Background/Objectives:** Pain is highly prevalent among hospitalized patients, and suboptimal pain assessment and management remain common in clinical practice. Nurses are central to timely pain recognition and intervention, yet knowledge and attitudinal gaps can hinder evidence-based pain care. Therefore, this study aimed to evaluate the impact of an evidence-based instructional program on nurses’ knowledge and attitudes toward pain management at a tertiary hospital in Saudi Arabia. **Methods:** A one-group pretest–posttest quasi-experimental study was conducted at King Fahad Military Medical Complex, Dhahran, Saudi Arabia (January–July 2025). Registered nurses providing direct patient care (N = 226) completed a researcher-developed questionnaire assessing pain management knowledge (30 items) and attitudes (10 items, 5-point Likert scale) immediately before and one week after a structured three-hour evidence-based educational program. Data were analyzed using descriptive statistics, paired-sample t-tests, and Pearson correlation coefficients (SPSS v30), with *p* < 0.05 considered statistically significant. **Results:** Baseline findings indicated moderate knowledge (mean of total scores = 15.54 ± 4.32) and generally positive attitudes toward pain management (mean = 3.83 ± 0.60). Knowledge scores increased significantly after the intervention to become moderate to high (pretest: 15.54 ± 4.32 vs. posttest: 18.65 ± 3.83; *p* < 0.001). Attitude scores also improved significantly following the program (*p* < 0.001). Knowledge and attitudes showed a significant positive correlation both preintervention (*r* = 0.241, *p* < 0.001) and postintervention (*r* = 0.435, *p* < 0.001). **Conclusions:** A brief evidence-based educational program yielded measurable improvements in nurses’ pain management knowledge and attitudes. Integrating structured pain education into continuing professional development may strengthen patient-centered pain care and support more consistent evidence-based practice in tertiary settings.

## 1. Introduction

Pain is one of the most common and distressing symptoms hospitalized patients experience and remains a major determinant of recovery, patient satisfaction, and overall quality of care [1,2]. When inadequately assessed or managed, it is associated with delayed healing, prolonged hospital stay, increased health-care costs, and reduced trust in health-care providers [3]. Despite advances in analgesic therapies and international clinical guidelines’ availability, effective pain management continues to be inconsistently achieved across health-care systems worldwide [4,5].

Nurses play a central role in pain management because they are responsible for ongoing pain assessment, documentation, analgesic administration, and patient response evaluation [6]. Their knowledge, attitudes, and beliefs directly influence clinical decision-making and the quality of pain-related care delivered to patients [7]. However, evidence from diverse clinical settings indicates persistent gaps in nurses’ knowledge and attitudes toward pain assessment and management [8,9]. Misconceptions surrounding opioid use, fear of addiction, underestimation of patient self-reporting, and uncertainty about appropriate dosing and reassessment are frequently reported barriers [10,11,12].

The literature presents diverging findings regarding nurses’ pain management preparedness. Some studies report moderate-to-acceptable levels of knowledge and generally positive attitudes [13,14], whereas others document substantial deficiencies and negative perceptions hindering optimal pain care [15,16,17]. Differences in educational background, clinical exposure, institutional culture, and access to continuing professional development may explain these inconsistencies. Systematic reviews and meta-analyses consistently suggest that targeted educational interventions can significantly improve nurses’ knowledge, attitudes, and pain-related practices [18,19,20]. Nonetheless, these improvements’ magnitude and sustainability vary across contexts, highlighting the need for setting-specific evaluations.

In Saudi Arabia, improving patient safety and quality of care is a national priority aligned with ongoing health-care transformation initiatives and Vision 2030 [21,22]. Several studies have examined nurses’ pain management knowledge and attitudes in general hospital settings. However, most have been descriptive, with limited evidence on structured educational interventions designed to improve pain management competencies [22,23]. In particular, intervention-based studies within tertiary military hospitals are limited [24,25]. These institutions serve unique patient populations and operate within highly structured clinical environments, which may influence both educational needs and practice patterns. Given these contextual complexities, evaluating the effectiveness of structured, evidence-based pain education in this setting is critical to inform targeted professional development and optimize patient-centered pain care.

Accordingly, the present study aimed to evaluate an evidence-based instructional program’s impact on nurses’ knowledge and attitudes toward pain management at a tertiary military hospital in Saudi Arabia. The study is expected to demonstrate that a brief, structured educational intervention can lead to statistically significant improvements in both knowledge and attitudes. This would reinforce the value of continuing education as a mechanism for strengthening patient-centered pain care and supporting evidence-based nursing practice.

## 2. Materials and Methods

### 2.1. Research Design

This study employed a quantitative, one-group pretest–posttest quasi-experimental research design to evaluate the effectiveness of an educational program on nurses’ knowledge and attitudes regarding pain management. This design enables change assessment by comparing participants’ scores before and after the intervention.

### 2.2. Setting

The study was conducted at King Fahad Military Medical Complex (KFMMC) in Dhahran, Kingdom of Saudi Arabia, from January 2025 to July 2025. This setting was selected because KFMMC is one of the largest tertiary care hospitals in the Eastern Province, providing comprehensive inpatient and outpatient services, including units where nurses routinely assess and manage patients’ pain. The size and diversity of the hospital’s units provided an ideal environment for recruiting nurses from diverse backgrounds and varying clinical experiences.

### 2.3. Participants and Sampling

To determine an adequate sample size for this study, standard sample size estimation parameters for survey-based research were applied. A minimum sample of approximately 214 nurses was deemed sufficient to achieve a 90% confidence level with a 5% margin of error for a target population of nearly 1000 nurses employed at KFMMC. This threshold ensured adequate statistical power to detect meaningful associations. However, the final sample comprised 226 nurses, exceeding the minimum recommended sample size. Convenience sampling was used to recruit participants. All registered nurses working at KFMMC who met the inclusion criteria were invited to participate. The respective head nurses distributed invitations to participate, including the study information sheet and informed consent form, through official hospital email channels.

The study targeted nurses providing direct patient care across all clinical departments within KFMMC. Nurses of different nationalities, qualifications, genders, and years of clinical experience were eligible for inclusion. Nurses in purely administrative or managerial roles, those working exclusively in outpatient clinics, and those on leave during data collection were excluded. Only nurses who voluntarily agreed and provided electronic informed consent were enrolled.

### 2.4. Data Collection Tools

To measure the knowledge and attitudes of nursing staff at the hospital, the researchers constructed a questionnaire based on the recent literature that included many references and websites. One of the essential references used to construct the data collection tool was the KnowPain-50 tool [26]. The original English version of the tool was used because the target population consisted of nurses at hospitals, and they all had good English communication skills. The study tool had three parts. Part I collected sociodemographic data and comprised 10 items, including age, gender, marital status, nationality, level of nursing education, years of professional experience, clinical unit or workplace, and prior training in pain assessment and management. Part II assessed nurses’ knowledge and consisted of 20 multiple-choice questions and 10 true/false questions. The knowledge section covered key domains, including pain physiology, pain assessment tools, and pharmacological and nonpharmacological pain management. Each correct response was assigned one point, and each incorrect response was assigned zero, yielding a total possible score of 0–30. A score of less than 12 indicated low knowledge (<40%), 12–<18 indicated moderate knowledge (40–<60%), and 18–30 (≥60%) indicated high knowledge. This cut score was identified based on the literature from previous studies [27,28].

Part III assessed nurses’ attitudes using 10 statements measured on a 5-point Likert scale (1 for “Strongly Disagree” and 5 for “Strongly Agree”). Items 6, 7, 8, and 10 were stated negatively, so the scores were reversed. The mean attitude score ranged from 1 to 5. The higher the mean score was, the more positive were nurses’ attitudes toward pain management.

In the present analyses, nurses’ knowledge was treated as a total score (range 0–30), whereas attitudes were analyzed as a mean score (range 1–5); all pre- and post-intervention comparisons were conducted using these score formats.

### 2.5. Validity of the Tool

Content validity was established through expert evaluation. Three clinical and academic nursing experts with pain assessment and management experience reviewed the instrument to assess its relevance, clarity, and alignment with the study objectives. Each item was evaluated for appropriateness, comprehensiveness, and conceptual consistency. The Content Validity Index was calculated, and S-CVI (average) was found to be 0.91 for the overall scale, reflecting a high degree of consensus among experts regarding the questionnaire’s overall validity. Based on their feedback, minor wording revisions were made to improve clarity. However, no items were added or removed. This process enhanced the instrument’s content validity and supported the study’s methodological rigor.

### 2.6. Reliability of the Tool

Instrument reliability was assessed prior to complete data collection using the test–retest method. The questionnaire was administered twice to a group of 21 nurses with a one-week interval between administrations. The consistency of responses demonstrated stable instrument performance. Internal consistency reliability was further examined using Cronbach’s alpha, yielding an average coefficient of 0.83 (0.88 for knowledge and 0.78 for the attitudes scale). This indicated acceptable internal reliability and cohesion among the questionnaire items. Internal consistency reliability was assessed using Cronbach’s alpha because all knowledge items were scored dichotomously (correct/incorrect). In this context, alpha is mathematically equivalent to the Kuder–Richardson formulas (e.g., KR-20). KR-20 may also be applied in future studies using similar dichotomous knowledge scales.

### 2.7. Pilot Study

A pilot study was conducted on 10% of the intended sample (approximately 21 nurses) to evaluate the instruments’ clarity, feasibility, and applicability. The pilot indicated that the tools were clear and practical for use in the clinical setting. No radical changes were made to the tool. However, nurses who participated in the pilot study were excluded from the main sample.

### 2.8. Data Collection Procedure

#### 2.8.1. Phase 1: Baseline Assessment (Pretest)

Following IRB approval and coordination with the nursing administration, the pretest questionnaire was administered to all selected nurses via simple random sampling. Participants were provided with the study information sheet and electronic informed consent form before they completed the survey. Baseline data were collected using a three-part instrument that measured sociodemographic characteristics, knowledge, and attitudes toward pain assessment and management. This phase established preintervention scores for subsequent comparison.

#### 2.8.2. Phase 2: Educational Intervention

After completing the baseline assessment, all participating nurses received a structured three-hour evidence-based educational program on pain assessment and management. The program was delivered in KFMMC’s designated educational facilities using the same environment and conditions for all sessions to maintain consistency.

The educational content was organized into five instructional sessions covering the following domains:

Fundamentals of Pain—Definition, causes, pathophysiology, acute vs. chronic pain, and factors influencing pain perception.

Pain Assessment—Nurse’s role, components of a comprehensive assessment, assessment methods, and validated pain assessment tools.

Pain Management Strategies—Goals of pain management, pharmacologic and nonpharmacologic interventions, and principles of safe analgesic administration.

Clinical Application—Nursing assessment and management of acute and chronic pain across different patient populations.

Evaluation of Pain Management—Documentation, reassessment principles, and evaluation of analgesic effectiveness.

Multiple teaching modalities were employed, including face-to-face lecture-based instruction, group discussions, and video-based demonstrations, to enhance comprehension and engagement.

#### 2.8.3. Phase 3: Postintervention Assessment (Posttest)

One week following the educational program, the same questionnaire used in Phase 1 was readministered to all participants who completed the pretest. This posttest enabled measurement of changes in knowledge and attitudes that were attributable to the intervention. Responses were matched with pretest data to allow paired comparison.

### 2.9. Ethical Considerations

The Institutional Review Board at KFMMC approved this study (AFHER-IRB-2024-028). All participants’ anonymity, confidentiality, and privacy were fully ensured. Participation in the study was voluntary, and participants had the right to withdraw at any time without penalty. A consent form was obtained from all participants prior to data collection, and all methods were conducted in accordance with relevant ethical guidelines and institutional regulations.

### 2.10. Data Analysis

Data were entered, cleaned, and analyzed using the Statistical Package for the Social Sciences (SPSS), Version 30 (IBM Corp., Armonk, NY, USA). Descriptive statistics were computed to summarize the study participants’ characteristics. Frequencies and percentages were used to describe categorical variables, including gender, age group, marital status, nationality, educational level, clinical unit, years of nursing experience, and previous exposure to pain assessment and management training. Means, standard deviations, and minimum and maximum values were calculated for the continuous outcome variables of knowledge and attitude scores.

To evaluate the effect of the educational intervention, paired-sample t-tests were conducted to compare pretest and posttest mean scores on nurses’ knowledge and attitudes toward pain management. To examine the relationship between nurses’ knowledge and attitudes, Pearson’s correlation coefficients were calculated. This analysis examined the strength and direction of the linear association between the two continuous variables. All statistical tests were two-tailed, and a *p*-value of <0.05 was considered statistically significant.

## 3. Results

### Demographic Variables

**Figure 1:**
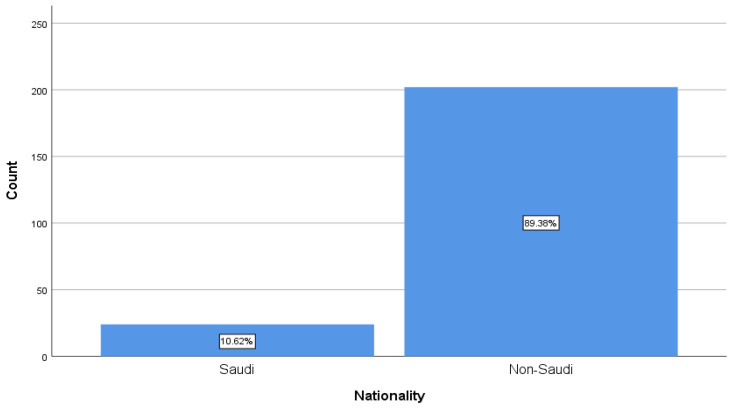
Distribution of participants based on nationality.

**Figure 2:**
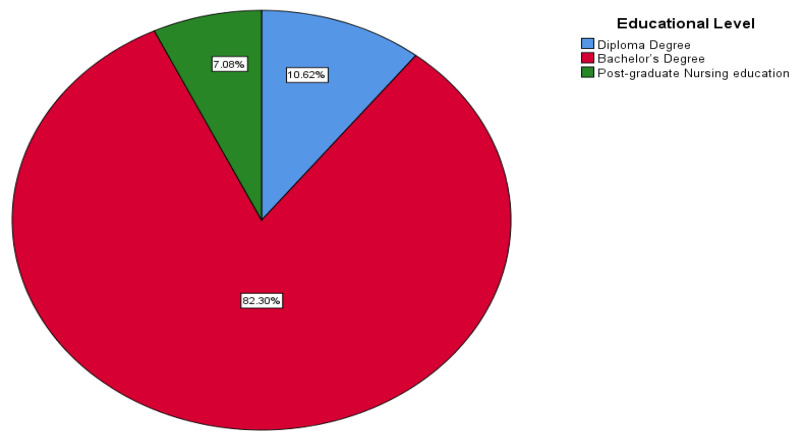
Educational level of participants.

**Figure 3:**
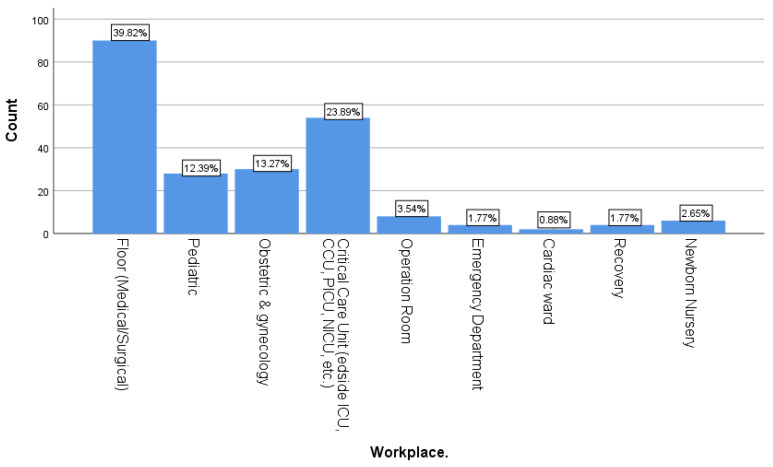
Distribution of participants based on working units.

The study included 226 nurses; the majority of nurses at KFMMC were female (97.3%) and non-Saudi (89.4%). Most participants were aged 35–39 (34.5%) and 30–34 (25.7%) years. Regarding marital status, nearly three-fifths (59.3%) were married, whereas 38.9% were single. Nationality data showed that the nursing workforce was overwhelmingly expatriate, with 89.4% of nurses non-Saudi and only 10.6% Saudi (Figure 1). In terms of education, most nurses held a bachelor’s degree (82.3%), with only 10.6% holding a diploma and 7.1% having postgraduate nursing education (Figure 2). The majority also had substantial professional experience—59.3% had worked for 10 years or more, whereas just 2.7% had less than one year of experience. For workplace distribution, the largest proportion of nurses worked in medical or surgical wards (39.8%), followed by critical care units (23.9%). Other areas, such as obstetrics and gynecology (13.3%), pediatrics (12.4%), and various specialty units (combined < 10%), were less represented (Figure 3).

Most nurses reported having strong pain management support in their setting. Almost all worked in hospitals with a pain management unit (97.3%) and stated that they received sufficient continuous education on pain assessment and management (95.6%). Additionally, the vast majority (80.5%) were exposed to a specialized educational program about pain management (Table 1).

Nurses’ knowledge of pain management improved on most items after the educational program, with higher proportions of correct answers across many assessment, pharmacological, and documentation questions. Notable gains were observed in knowledge of the WHO pain ladder update (15.5–34.5%), the definition and nature of pain (43.4–63.7%), appropriate use of morphine and other analgesics (e.g., severe pain management, 58.8–76.1%; preadministration assessment for IV morphine, 66.4–87.6%), and key practice guidelines such as documentation of pain medications (77.9–97.3%) and recognition of indicators of chronic pain (74.8–91.2%). Overall, the program appears to have strengthened nurses’ pain management knowledge, particularly in critical clinical decision-making and best-practice procedures.

Nurses’ overall knowledge scores significantly improved after the educational program, as indicated by a paired-samples t-test. Nurses’ total knowledge scores increased notably from pre-test to post-test. The mean score rose from 15.54 (SD = 4.32) before the educational program to 18.65 (SD = 3.83) afterward, indicating higher knowledge levels overall. The paired-samples *t*-test showed a statistically significant mean difference of −3.12 points (t (225) = −8.29, *p* < 0.001), confirming a significant improvement in nurses’ knowledge following the program with a moderate effect size (Cohen’s d ≈ −0.55). This indicated that the program had a meaningful moderate positive impact on nurses’ pain management knowledge (Table 2).

Nurses generally held favorable attitudes toward pain management both before and after the educational program, with mean scores close to “agree” or higher on most positively worded items (e.g., recognizing surgical pain as severe, empathizing with patients, feeling comfortable assessing and medicating pain). After the program, small improvements were evident in several positive attitudes, such as greater comfort in pain assessment and medication administration (mean 4.16–4.49) and stronger empathy and functional assessment in pain evaluation (e.g., empathizing with postoperative patients, 4.04–4.24; assessing function, 4.05–4.24). Items reflecting negative or less desirable attitudes (e.g., irritation with frequent requests, ignoring patients frequently asking for analgesics, restraining aggressive patients) showed higher mean scores, which, depending on the coding direction, indicated persistent problematic beliefs and increased disagreement with the reverse-coded items (Table 3).

Nurses’ overall attitudes toward pain management improved significantly after the educational program, as indicated by a paired-samples *t*-test (Table 4). The mean attitude score increased from 3.83 (SD = 0.60) before the intervention to 4.07 (SD = 0.53) afterward. The paired-samples *t*-test showed that this mean increase of 0.24 points was statistically significant (t (225) = −4.48, *p* < 0.001), with a small-to-moderate effect size (Cohen’s d = −0.30) indicating a significant small-to-moderate improvement in nurses’ attitudes following the program and shift toward more favorable attitudes.

The Pearson correlation coefficient test revealed a shift from a weak positive correlation between knowledge and attitude before the intervention (r = 0.241, *p* < 0.001) to a moderate positive correlation after it (r = 0.435, *p* < 0.001). Overall, combining pre- and post-test data showed a moderately significant correlation (r = 0.373, *p* < 0.001), suggesting that participants with higher knowledge consistently displayed more positive attitudes, particularly after the educational program.

**Figure 4:**
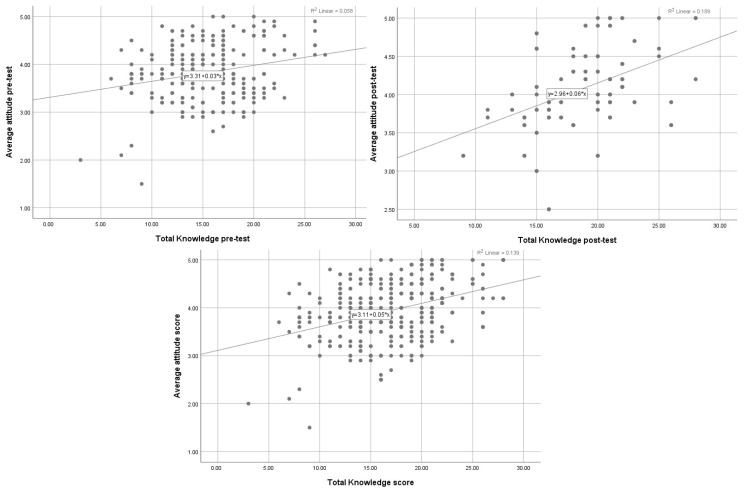
Correlation between nurses’ knowledge and attitudes toward pain management. The gray dots represent individual participants’ data points in the scatterplots.

Figure 4 displays three scatterplots illustrating a positive linear relationship between nurses’ pain management knowledge scores and their average attitude scores at pretest and posttest, and when pretest–posttest knowledge scores were combined. In all three panels, higher total knowledge scores were associated with more favorable attitudes toward pain management, and this correlation became more positive in the post-test. In the figure, this is indicated by the upward regression lines and a moderate proportion of explained variance (*R*^2^ ≈ 0.139, *p* < 0.001).

## 4. Discussion

This study examined nurses’ knowledge and attitudes toward pain management and evaluated the impact of a brief, evidence-based educational intervention in a tertiary military hospital in Saudi Arabia. The findings demonstrated that nurses entered the program with moderate baseline knowledge and generally positive attitudes toward pain management and that participation in a structured three-hour instructional program resulted in statistically significant improvements in both domains. High-level mean knowledge scores and more positive attitudes toward pain management were obtained. Further, a consistently positive correlation was observed between knowledge and attitudes before and after the intervention. These findings supported the working hypothesis that targeted education may strengthen nursing pain management’s cognitive and affective foundations.

The baseline finding of moderate knowledge aligned with several regional and international studies’ reports of comparable levels of pain-related knowledge among nurses working in tertiary care settings [29,30,31]. Previous research has shown that nurses often possess a foundational understanding of pain physiology and basic management principles. However, gaps persist in more complex areas such as opioid pharmacology, equianalgesic dosing, and systematic pain reassessment [32,33]. This pattern suggests that the challenge in pain management is not solely a lack of knowledge but also variability in depth, consistency, and clinical application. In highly demanding environments, such as tertiary and military hospitals, systemic factors—including workload, time constraints, and hierarchical decision-making—may further limit the translation of knowledge into practice [23,34].

The observed improvement in knowledge following the educational intervention was consistent with a robust body of evidence demonstrating the effectiveness of structured pain education programs [35,36,37]. Meta-analyses and quasi-experimental studies across diverse clinical contexts have repeatedly shown that even short, focused educational interventions can yield significant gains in nurses’ pain-related knowledge [38]. Although the absolute increase in mean knowledge scores in the present study was modest, it was statistically significant and clinically relevant, particularly given the relatively high baseline scores. The effect size analysis further supported the practical importance of this improvement. The educational program demonstrated a moderate effect size on knowledge (Cohen’s d ≈ 0.55), suggesting that the intervention produced a meaningful enhancement in nurses’ understanding of pain assessment and management principles rather than a trivial statistical change. This finding suggested that targeted reinforcement of evidence-based principles can refine and consolidate existing knowledge, rather than merely addressing fundamental deficits.

Nurses in this study also demonstrated generally positive baseline attitudes toward pain management, an encouraging finding given the documented influence of attitudes on clinical behavior [39]. Positive attitudes are associated with greater advocacy for patients, more consistent pain assessment, and increased responsiveness to analgesic needs [40]. However, previous studies have obtained mixed findings, with several of them reporting predominantly negative or ambivalent attitudes among nurses, often driven by concerns about opioid addiction, tolerance, or the subjectivity of pain reports [41,42,43]. The comparatively favorable attitudes observed in the current study may reflect prior exposure to pain education, institutional emphasis on patient-centered care, or accumulated clinical experience within the study setting.

The educational intervention produced a statistically significant improvement in nurses’ attitudes toward pain management. The effect size for attitudes was small to moderate (Cohen’s d ≈ 0.30), indicating that although the numerical increase in the mean attitude score was relatively small (0.24 points on a 5-point scale), the change reflects a meaningful shift toward more positive attitudes regarding pain management among participating nurses. Such improvements in nurses’ knowledge and attitudes are particularly important because they are closely linked to clinical decision-making, pain assessment practices, and timely analgesic administration, ultimately contributing to improved patient comfort and safety in clinical settings. This finding supported the growing recognition that education can influence knowledge acquisition and the beliefs and perceptions shaping clinical decision-making [44,45]. Addressing attitudinal barriers—such as fear of opioid-related harm or skepticism toward patient self-report—is essential for achieving meaningful improvements in pain care. The results suggested that evidence-based education, particularly when it explicitly addresses misconceptions and ethical dimensions of pain management, can foster more confident and patient-centered attitudes among nurses.

The significant positive correlation between knowledge and attitudes observed both before and after the intervention highlighted the interdependent relationship between these constructs. This relationship suggested that greater knowledge may enhance nurses’ confidence and openness toward proactive pain management, whereas positive attitudes may, in turn, motivate engagement with evidence-based practices and continued learning [46,47]. These findings aligned with those of previous studies’ showing that nurses with higher knowledge levels tend to report more favorable attitudes and greater self-efficacy in pain management [48]. From a theoretical perspective, these results supported models of clinical competence that emphasize integrating cognitive, affective, and behavioral domains.

Taken together, the findings underscored the value of educational interventions as a strategy for strengthening nursing contributions to pain management. However, education alone may be insufficient to ensure sustained improvements in practice. The modest magnitude of change observed, particularly in attitudes, suggested that ongoing reinforcement, mentorship, and supportive organizational structures are necessary to translate gains in knowledge and attitudes into consistent clinical behavior [49,50]. Embedding pain education into continuous professional development programs and aligning it with institutional policies and interdisciplinary collaboration may enhance long-term impact.

Several implications for future research emerged from this study. Longitudinal studies are needed to examine the sustainability of educational effects over time and their influence on actual pain management practices and patient outcomes [51]. Comparative studies using controlled or randomized designs could further clarify the effectiveness of different educational formats, durations, and delivery methods [52]. Additionally, qualitative research exploring nurses’ experiences and perceived barriers to pain management could provide deeper insight into contextual factors influencing practice, particularly in specialized settings such as military hospitals [53].

This study has several limitations. For example, it was designed as a single-group pretest–posttest study because of the limited number of staff nurses available at the study site. Consequently, the absence of a comparison group may have limited the ability to attribute observed changes solely to the intervention and affected the internal validity of the findings. Additionally, external factors, such as clinical experience gained during the study period and potential test–retest effects, may have influenced the observed outcomes. The instrument was author-developed and does not have full psychometric validation. The postintervention assessment was conducted only one week after the educational program, limiting conclusions regarding long-term knowledge retention and sustained attitudinal change. A convenience sampling approach was used, which may have limited the sample’s representativeness and reduced the generalizability of the findings. Voluntary participation may also have introduced selection bias. Furthermore, data were collected from a tertiary military hospital with a predominantly non-Saudi nursing staff, further limiting the generalizability of the results to other health-care settings.

In summary, this study contributes to the growing evidence that evidence-based educational interventions can positively influence nurses’ knowledge and attitudes toward pain management. By situating these findings within the broader literature, the study highlights both the promise and limitations of education-focused strategies and reinforces the need for multifaceted approaches to improving pain care in complex clinical environments [54].

## 5. Conclusions

This study demonstrates that nurses working in a tertiary military hospital possess a solid foundational level of knowledge and generally positive attitudes toward pain management. The findings show that a brief, structured, evidence-based educational program can produce statistically significant improvements in both knowledge and attitudes, even within a workforce that has substantial clinical experience and prior exposure to pain-related education.

The observed positive correlation between knowledge and attitudes highlights the interrelated nature of these domains and underscores the importance of addressing both simultaneously when designing educational interventions. Enhancing nurses’ understanding of pain assessment and management appears to reinforce more patient-centered, confident, and proactive attitudes, essential for high-quality pain care.

The results are clinically meaningful and suggest that continuous, targeted education can refine existing competencies and help address persistent misconceptions. To achieve sustained improvements in practice, such educational efforts should be embedded within ongoing professional development frameworks and be supported by organizational policies facilitating evidence-based pain management.

Overall, the study reinforces the value of structured pain education as a practical and effective strategy for strengthening nursing practice and supporting consistent, high-quality pain management in complex clinical environments.

## Figures and Tables

**Table 1:** Pain Management Resources and Educational Exposure Among Nurses.

Pain Management Facilities	No	Yes
	*n* (%)	*n* (%)
Do you have a pain management unit in your hospital?	6 (2.7%)	220 (97.3%)
Have you been exposed to a specialized educational program about pain management?	44 (19.5%)	182 (80.5%)
Do you receive enough continuous education about pain assessment and pain management in your hospital?	10 (4.4%)	216 (95.6%)

**Table 2:** Difference in nurses’ knowledge before and after the educational program.

Paired Samples Statistics		Mean	N	Std. Deviation	Std. Error Mean				
Pair 1	Total Knowledge pre-test	15.5398	226	4.32160	0.28747				
	Total Knowledge post-test	18.6549	226	3.83381	0.25502				
Paired Samples Test		Paired Differences					t	df	Sig. (2-tailed)
		Mean	Std. Deviation	Std. Error Mean	95% Confidence Interval of the Difference				
					Lower	Upper			
Pair 1	Total Knowledge pre-test	−3.11504	5.65175	0.37595	−3.85588	−2.37421	−8.286	225	0.000 **
	Total Knowledge post-test								

** Significant at less than 0.01.

**Table 3:** Nurses’ attitudes toward pain management before and after the educational program.

Nurses’ Attitudes	Before Educational Program		After Educational Program	
	Mean	SD	Mean	SD
1. In my opinion, surgical pain is severe pain that needs immediate intervention.	4.40	0.948	4.45	0.904
2. A calm patient who complains of moderate pain in the chest tube insertion site should be immediately given pain medication as ordered.	3.97	0.975	4.05	0.852
3. I believe patients who undergo major surgery feel severe pain that needs round-the-clock pain medication.	4.37	0.916	4.41	0.738
4. I empathize with patients who complain of pain on their postoperative site.	4.04	0.947	4.24	0.643
5. I can assess function and activity status in pain assessment with careful questioning.	4.05	0.930	4.24	0.734
6. Frequent high pain scores indicate a patient is exaggerating.	2.66	1.208	2.96	1.302
7. I feel irritated with patients who frequently ask for pain medication.	3.35	1.557	3.85	1.156
8. A patient who is frequently asking for pain medication should just be ignored.	3.82	1.277	4.13	1.002
9. I am comfortable in assessing pain and giving pain medication as ordered.	4.16	0.929	4.49	0.583
10. In my opinion, the best way to calm an aggressive patient who complains of severe pain is to restrain them as ordered.	3.49	1.370	3.90	1.258

**Table 4:** Difference in nurses’ attitudes before and after the educational program.

Paired Samples Statistics		Mean	N	Std. Deviation	Std. Error Mean				
Pair 1	Average attitude pre-test	3.8314	226	0.59773	0.03976				
	Average attitude post-test	4.0717	226	0.52738	0.03508				
Paired Samples Test		Paired Differences					t	df	Sig. (2-tailed)
		Mean	Std. Deviation	Std. Error Mean	95% Confidence Interval of the Difference				
					Lower	Upper			
Pair 1	Average attitude pre-test	−0.24027	0.80571	0.05360	−0.34588	−0.13465	−4.483	225	0.000 **
	Average attitude post-test								

** Significant at less than 0.01.

## Data Availability

The data presented in this study are available from the corresponding author upon reasonable request. The data are not publicly available due to institutional and ethical restrictions.

## Abbreviations

The following abbreviations are used in this manuscript:

KFMMC	King Fahad Military Medical Complex
IRB	Institutional Review Board
SPSS	Statistical Package for the Social Sciences
APC	Article Processing Charge
